# The History and Capabilities of Herbal Simples

**Published:** 1890-12-20

**Authors:** W. T. Fernie


### The History and Capabilities of Herbal Simples.

By W. T. Fernie, M.D.

### XXIV.—CRESS.

The Cress of the herbalist is a noun of multitude; it comprises several sorts, differing in kind, but possessing the common properties of wholesomeness and pungency. As Pope sings, "Here order in variety we see; and here, though all things differ, all agree." Each kind of cress belongs to the cruciferous order of plants; whence, perhaps, comes the common name. But this is thought rather by certain botanists to be derived from the Latin verb, *crescere*—to increase, or grow fast—whilst others refer the term "cress" to its Greek analogue, *cardamon*, a fortifier of the heart. The several varieties of cress are stimulating and antiscorbutic, whilst each contains a particular essential principle, of acrid flavour, and of sharp, biting qualities. The whole genus of cresses is styled lepidium, or scaly, with reference to the shape of the seed-pouches. It includes land cress (formerly dedicated to Saint Barbara), broad-leaved cress, or "the poor man's pepper," penny cress (thlapsus), garden or town cress, and the well-known edible water cress. But I shall consider the herb only in the two last of these sorts, "one foot in sea and one on shore," like Shakespeare's *men*, for whom ladies were to sigh no more, because the faithless males were deceivers ever. Formerly the Greeks attached much value to the whole tribe of cresses, which they esteemed as very beneficial to the brain. A favourite maxim with them was, "Eat cresses, and get more wit."

In England the plants have been long cultivated artificially, and often as a source of profit. Hence arose the saying that a graceless fellow is not worth a curse, or "cress" (in German "kers"). So Chaucer said about a character in the Canterbury Tales, "Of paramours ne fraught he not a kers." But some writers have thought this proverb to refer rather to the wild cherry, or kerse, and thus to be identical in meaning with our modern saying, "not worth a fig." William Coles, in his "Paradise of Plants," 1650, says, "I shall reckon up the chief sorts of the herb as Garden cresses, Land cresses, Winter cresses, and the impatient Lady Smock, which flowereth in January. The Garden cress, being green, and therefore the more qualified by reason of its humidity, is eaten by country people either alone, with butter, or with lettuce, and Parslane, in Sallets or otherwise. The Winter cress is used, as other cresses and Rockets in summer, so this in winter, with as great desire and content to be eaten, when variety of Sallets are not to be had." These aromatic herbs were employed to season the homely dishes of our forefathers before commerce had brought the spices of the East at a cheap rate to our doors; and cresses were then used commonly by peasants for the same purpose. The black or white pepper of to-day was so costly that "to promise a saint yearly a pound of it was considered a liberal bequest." Therefore, the leaves of wild cresses were held to be a sufficient substitute for giving pungency to food. Remarkable amongst them was Dittander-sativum, a species found chiefly near the sea, with foliage so hot and acrid that the plant went then by the name of "poor man's pepper." Perhaps Peter Piper of past celebrity then picked a peck of this pickled condiment. But, if so, where's the peck now which Peter Piper picked? "The herbe," says Lyte, "is fondly and unlearnedly called in English Dittany. It were better, in following the Dutchmen, to call it Pepperwurt." Pliny saith, "It is of the number of scorching and blist'rin simples."

*The Garden or Town Cress*—called sativum, from satum, a pasture—is the sort commonly associated with the herb mustard in our "mustard and cress." It has been grown in

England since the middle of the sixteenth century; and the name Town cress which it bears was given because of its cultivation in "tounes," or enclosures, as the word "tun" formerly meant a close or pasture in connection with farm buildings. Garden cress was also termed passerage—passer, to drive away rage, madness—because of its supposed power to expel hydrophobia. It contains sulphur and a special ardent medicinal volatile oil. Its small leaves, combined with those of our white garden-mustard, are much esteemed as a salad, and are excellent against rheumatism and gout. Likewise it is a preventive of scurvy, by reason of its mineral salts. In these beneficial respects the twin plants mustard and cress are happily consorted, and well play a capital common part, like the "two single gentlemen rolled into one," of George Colman, the younger. Lord Bacon distinguished this and other cresses as plants friendly to life.

*(To be continued.)*

